# Identification of an eight-gene signature for survival prediction for patients with hepatocellular carcinoma based on integrated bioinformatics analysis

**DOI:** 10.7717/peerj.6548

**Published:** 2019-03-20

**Authors:** Guo-jie Qiao, Liang Chen, Jin-cai Wu, Zhou-ri Li

**Affiliations:** 1 Institute of Tropical Agriculture and Forestry, Hainan University, Hainkou, China; 2 Department of Hepatobiliary Surgery, Hainan Provincial People’s Hospital, Hainan Medical College, Hainkou, China

**Keywords:** Hepatocellular carcinoma, Gene signature, Prognostic marker, Overall survival

## Abstract

**Background:**

Hepatocellular carcinoma (HCC) remains one of the leading causes of cancer-related death worldwide. Despite recent advances in imaging techniques and therapeutic intervention for HCC, the low overall 5-year survival rate of HCC patients remains unsatisfactory. This study aims to find a gene signature to predict clinical outcomes in HCC.

**Methods:**

Bioinformatics analysis including Cox’s regression analysis, Kaplan-Meier (KM) and receiver operating characteristic curve (ROC) analysis and the random survival forest algorithm were performed to mine the expression profiles of 553 hepatocellular carcinoma (HCC) patients from The Cancer Genome Atlas (TCGA) and Gene Expression Omnibus (GEO) public database.

**Results:**

We selected a signature comprising eight protein-coding genes (DCAF13, FAM163A, GPR18, LRP10, PVRIG, S100A9, SGCB, and TNNI3K) in the training dataset (AUC = 0.77 at five years, *n* = 332). The signature stratified patients into high- and low-risk groups with significantly different survival in the training dataset (median 2.20 vs. 8.93 years, log-rank test *P* < 0.001) and in the test dataset (median 2.68 vs. 4.24 years, log-rank test *P* = 0.004, *n* = 221, GSE14520). Further multivariate Cox regression analysis showed that the signature was an independent prognostic factor for patients with HCC. Compared with TNM stage and another reported three-gene model, the signature displayed improved survival prediction power in entire dataset (AUC signature = 0.66 vs. AUC TNM = 0.64 vs. AUC gene model = 0.60, *n* = 553). Stratification analysis shows that it can be used as an auxiliary marker for many traditional staging models.

**Conclusions:**

We constructed an eight-gene signature that can be a novel prognostic marker to predict the survival of HCC patients.

## Introduction

Hepatocellular carcinoma (HCC) is the predominant type of liver cancer and has an increasing worldwide prevalence (Bosch et al., 2004). In many countries, liver cancer mortality rates rise in accordance with HCC incidence rates, reflecting the poor survival of this cancer (Altekruse et al., 2014). With recent advances in therapeutic intervention of documented cases of HCC, such as liver transplantation, surgical resection locoregional therapies and chemotherapy, the 5-year survival of patients at early stage is higher than 50% (Yu et al., 2012), and the median overall survival is 60 months (Roessler et al., 2010). However, up to 70% of HCC patients undergoing resection or ablation experience tumour recurrence within 5 years (Roessler et al., 2012), and more than 70% of patients are unable to benefit from these interventions due to potential liver dysfunction and/or advanced disease performance. The median survival time of patients who suffer from unresectable disease is approximately 6–20 months, and their 5-year survival is less than 5% (Mikhail, Cosgrove & Zeidan, 2014). Therefore, discovering prognostic biomarkers to accurately predict clinical outcome is urgently needed for hepatocellular carcinoma patients.

Numerous attempts have been made to find survival prediction biomarkers and establish guidelines for HCC long-term prognosis. The potential markers of HCC prognosis reported in the literature are divided into the following categories: (1) single molecules as an independent prognostic indicator, such as serum alpha-fetoprotein (AFP), des-gamma-carboxy-prothrombin (DCP) or other novel markers that are currently being studied (Calderaro et al., 2017; Li et al., 2017); and (2) gene signature constructed by several to dozens of prognostic genes through analysing high-throughput gene expression profiles. With the development of sequencing and precision medicine, HCC survival related gene signatures have become a research hotspot. The Cancer Genome Atlas (TCGA) and Gene Expression Omnibus (GEO) public databases, with a broad range of hepatocellular carcinoma gene expression data, facilitate molecular analysis for HCC prognostic biomarker screening. However, auxiliary markers for the traditional staging models, such as TNM stage, T stage, and BCLC stage, are lacking.

In this study, we obtained the expression profiles of HCC patients from a large dataset in the TCGA and GEO database to construct a prognostic PCG signature, verified its prediction power for survival and then demonstrated its clinical roles with the existing staging models.

## Materials and Methods

### HCC patients and mRNA expression profiles

One dataset including gene expression profiles and associated corresponding clinical information of HCC patients analysed in this study was downloaded from The Cancer Genome Atlas (TCGA, http://cancergenome.nih.gov/). The other dataset from GSE14520 with mRNA expression profiling was obtained from the Affymetrix HT Human Genome U133A Array (Affymetrix, Santa Clara, CA, USA).

Gene exclusion criteria were as follows: genes with missing expression values in more than 30% of samples or patients and genes whose expression values were 0 in all samples. The k-nearest neighbour method was used to calculate the remaining missing gene expression values. As the data were from a public database, further approval by an ethics committee was not required. This study met the publication guidelines provided by TCGA (http://cancergenome.nih.gov/publications/publicationguidelines).

### Construction of a prognostic PCG signature in the training dataset

Univariate Cox proportional hazards regression analysis was performed to screen out those genes with a significant relationship with patients’ OS in the training dataset (Guo et al., 2018). Then, we used the random survival forests-variable hunting (RSFVH) algorithm to filter prognostic genes until twelve PCGs were screened out (Guo et al., 2016).

Subsequently, based on the above prognostic genes, we performed a multivariate Cox regression analysis and constructed the risk score model as follows:

\begin{eqnarray*}& & \mathrm{Risk~ Score} (RS)={\mathop{\sum \nolimits }\nolimits }_{i=1}^{N} ({\mathrm{Exp}}_{i} \ast  \mathrm{Co}{\mathrm{e}}_{i}) \end{eqnarray*}

where N is the number of prognostic PCGs, Exp_i_ is the expression value of PCGs, and Coe_i_ is the estimated regression coefficient of PCGs in the multivariate Cox regression analysis. Twelve PCGs could form 2^12^−1 = 4,095 combinations or signatures. The corresponding risk scores for the patients from both training and validation dataset were calculated using the risk score system. Receiver operating characteristic (ROC) curves were plotted based on the risk score and survival status of each patient. Then, we removed the signature with the maximum area under the curve (AUC) in the training dataset.

### Validation experiments in cell lines

We isolated total RNA from the normal (293T, L02) or cancer (Hep-G2, Hep-3B, PLC/PRF/5, Huh-7) cell lines with a QIAGEN RNeasy Mini Kit. Total RNA was then reverse-transcribed by 5 ×PrimeScript RT Master Mix according to the manufacturer’s recommendation. PCR was performed in the presence of 1 µl complementary DNAs (cDNAs), 0.5 µl forward and reverse primers and 2 ×Taq PCR Master Mix in a total volume of 20 µl. We used GAPDH (Forward: GGAGCGAGATCCCTCCAAAAT, Reverse: GGCTGTTGTCATACTTCTCATGG) as an internal reference. The reaction conditions were carried out according to the manufacturer’s instructions. PCR products were analysed on a 2% agarose gel.

### Statistical analysis

Kaplan–Meier (KM) curves were plotted when the median risk score in each dataset was used as the cutoff value to compare survival risk between high-risk and low-risk groups. Multivariate Cox regression analysis was performed to test whether the PCG signature was an independent prognostic factor. Significance was defined as *P* < 0.05. All were performed in R (R Core Team, 2018) with R packages pROC, survival, TimeROC and randomForestSRC, which were downloaded from Bioconductor.

Furthermore, the co-expressed relationships between the prognostic PCGs in the signature and all other protein-coding genes were computed using the Pearson test, and those genes with *P* value < 0.05 and absolute value of the Pearson coefficient > 0.5 were selected for Gene Ontology (GO) and Kyoto Encyclopaedia of Genes and Genomes (KEGG) enrichment analyses, which were performed with the clusterProfiler package (Yu et al., 2012).

## Results

### Patient characteristics and expression profiles

From the TCGA database, 423 HCC samples with mRNA expression profiles and clinical data were downloaded, including 50 normal tissues and 373 cancer tissues. Simultaneously, we obtained the mRNA expression profiles of 445 HCC samples from GSE14520 and their clinical characteristics, including 220 normal and 225 cancers. Then, 332 TCGA and 221 GEO cancer samples with corresponding overall survival data (OS) were selected as training and test sets to explore a prognostic PCG signature and validate the power of the signature in predicting the survival of HCC patients, respectively. After the initial analysis as described in the methods, a total of 16,101 PCG expression values of HCC patients were obtained. All these gene expression values were log_2_ transformed. Of the enrolled 553 HCC patients, the median age was 62 years (17–90 years), and 528 were stage I, II, III, and IV, while the stages of 25 patients were unknown. The clinical information of the two datasets is summarized in Table 1.

**Table 1:** Summary of patient demographics and clinical characteristics.

**Characteristic**	**TCGA (*n* = 332)**	GSE14520**(*n* = 221)**
Age (years)		
>62	170	33
≤62	162	188
Sex		
female	110	30
Male	222	191
Vital status		
Living	225	136
Dead	107	85

### Identification of prognostic mRNAs from the training dataset

**Figure 1:**
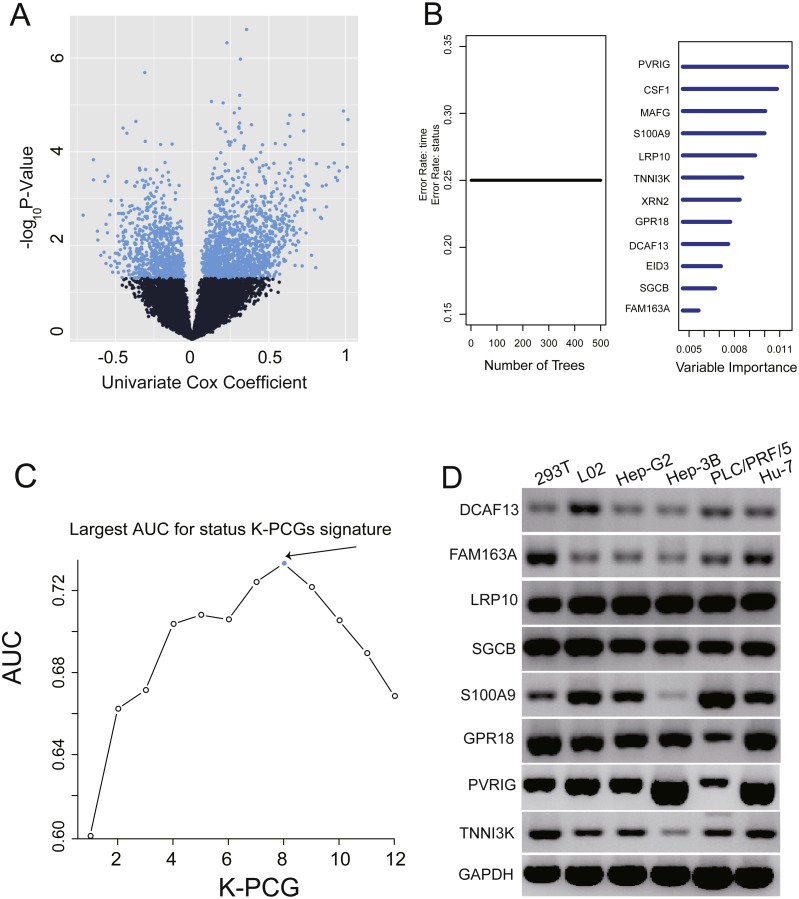
Identification of the prognostic PCG signature in the training dataset. (A) Volcano plot of the survival associated PCGs in univariate cox regression analysis. (B) According to important score to filter genes which were calculated by random survival forest analysis, the twelve genes with highest accuracies (*k* = 1, 2…12, *k* represents the gene number) are shown in the plot. (C) After calculating the AUC of 4,095 signatures, the prognostic PCG-lncRNA signature with biggest prediction power (*n* = 8) was screen out. (D) Validating the expression of the selected eight genes in six cell lines.

We conducted a univariate Cox proportional hazards regression analysis of the 16,101 PCGs in the training dataset and revealed a subset of 2,231 PCGs that was significantly correlated with patients’ OS (*P* value < 0.05). To display these selected genes, a volcano plot using the univariate Cox coefficient as the *X*-axis and −log_10_ (*P* value) as the *Y*-axis was constructed (Fig. 1A). As shown in Fig. 1A, we identified 2,231 genes with significant differences (*P* < 0.05), which are represented by blue dots; black dots represent the remaining genes with no statistically significant differences. Then, we further performed random forest supervised classification algorithm using the 2,231 genes and screened out 12 PCGs (PVRIG, CSF1, MAFG, S100A9, LRP10, TNNI3K, XRN2, GPR18, DCAF13, EID3, SGCB, and FAM163A) strongly related to patient survival according to the permutation important score by random survival forests-variable hunting (RSFVH) algorithm (Fig. 1B).

### Construction of the prognostic PCG signature in the training dataset

These 12 screened PCGs could construct 4,095 risk score models and form 4,095 signatures. To select a signature with the largest prediction power in the training dataset, we performed ROC analyses using patients’ survival status and signature risk scores as variables. Through comparing their areas under the respective ROC curves (AUC), a PCG signature comprising eight genes (PVRIG, S100A9, LRP10, TNNI3K, GPR18, DCAF13, SGCB, and FAM163A) with the max AUC was screened out (Fig. 1C, Table 2).

**Table 2:** Identities of PCGs in the prognostic signature and their univariable cox association with prognosis.

**Database ID**^a^	**Gene symbol**	**Gene name**	**Coefficient**^b^	***P***^b^	**Gene expression level association with prognosis**	**Primer (5′–3′)**
						**Forward(UP)/Reverse(down)**
ENSG00000164934	DCAF13	DDB1 and CUL4 associated factor 13	0.389	0.002	high	ACTGCACAGCTAAAGAACCG
						TCCCAGACTACTTCCAGTCAC
ENSG00000143340	FAM163A	Family with sequence similarity 163 member A	0.204	0.002	high	TTTTACATACGGACGGCTGACA
						CTAATAGCCCTTGGATTGGTGAA
ENSG00000197324	LRP10	LDL receptor related protein 10	0.533	<0.001	high	GCAGTGCTCTTAGAAGTGCAG
						CCTGATGGTGACAGTCTGTTC
ENSG00000163069	SGCB	Sarcoglycan beta	0.226	0.001	high	AGCAAAGTTCCAATGGTCCTG
						TCATCAATCGGAATGTATCCAGC
ENSG00000163220	S100A9	S100 calcium binding protein A9	0.202	<0.001	high	GGTCATAGAACACATCATGGAGG
						GGCCTGGCTTATGGTGGTG
ENSG00000125245	GPR18	G protein-coupled receptor 18	−0.311	0.001	low	CAGTTGTACCACCAAGAAGAG
						GCACTAATAAAGGCAAGAAGC
ENSG00000213413	PVRIG	PVR related immunoglobulin domain containing	−0.345	0.002	low	ACGTCCCTTATGCCACTATCA
						AGCGTAGAGTCCATTCTCAACA
ENSG00000116783	TNNI3K	TNNI3 interacting kinase	−0.48	0.002	low	TCATAAACATCAACCACCAAG
						TTCATAAGCCCACATCAAACA

**Notes.**

a Ensembl database.

b Derived from the univariable Cox regression analysis in the training set.

To validate the expression of these genes, we extracted total RNA from six cell lines (see ‘Methods’). PCR and agarose gel results using the eight gene primers shown in Table 2 revealed that these eight genes were highly abundant in normal and liver cancer-related cell lines (Fig. 1D).

### Performance evaluation of the PCG signature for survival prediction

**Figure 2:**
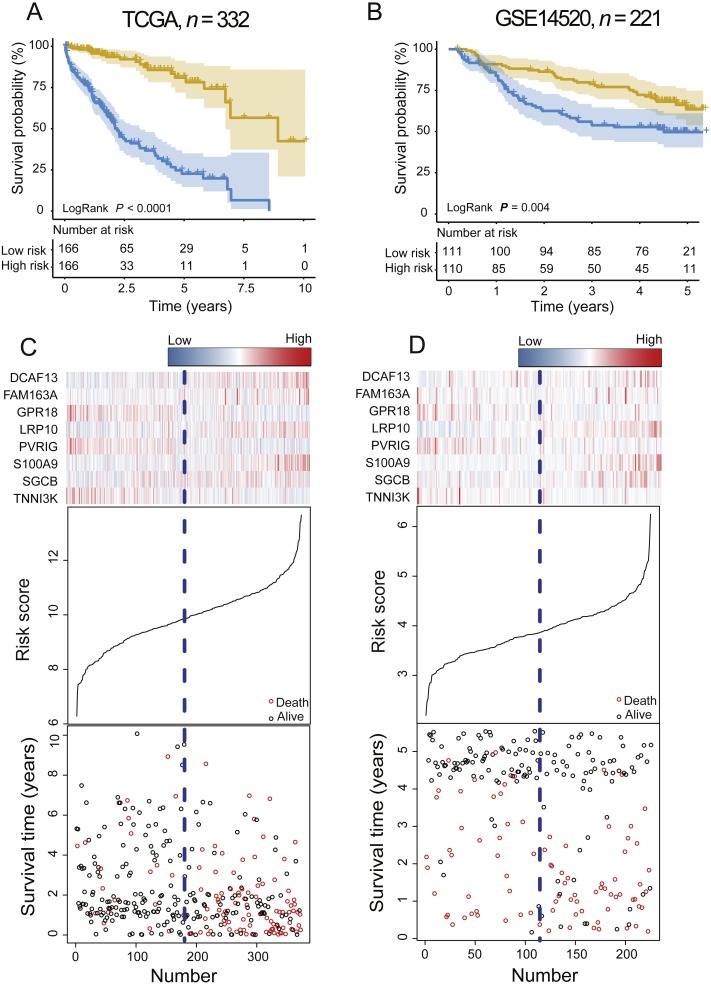
The PCG signature predicts overall survival of patients with HCC in the training set and test set. (A, B) Kaplan–Meier survival curves classify patients into high- and low-risk groups by the PCG signature in the training and test dataset. *P* Values were calculated by log-rank test. (C, D) Risk score distribution, survival status and gene expression patterns for patients in high- and low-risk groups by the PCG signature in the training and test dataset.

In the training dataset, with the median value of risk score as cutoff, patients were divided into a high-risk group (*n* = 166) and a low-risk group (*n* = 166). Kaplan–Meier survival analyses were performed to compare the overall survival of two groups of patients. The low-risk group had significantly better clinical outcomes than the high-risk group (2.20 vs. 8.93 years, log-rank test *P* < 0.001; Fig. 2A) in the training dataset. The OS rate of patients in the high-risk group was 32.25% and that of patients in the low-risk group was 75.60%.

To validate the prognostic prediction power of the signature, we applied the PCG signature-based risk model to the test set. The median risk scores of 221 HCCs were calculated as the cutoff point in the test set using the Affymetrix gene chip rather than RNA-seq. The test dataset was divided into high-risk and low-risk groups. Kaplan–Meier curves for the high- and low-risk groups in the test dataset are shown in Fig. 2B (median 2.68 vs. 4.24 years, log-rank test *P* = 0.004, *n* = 221).

The risk score, gene expression heat-map, and survival status of each HCC patient in the training or test cohort are plotted in Figs. 2C and 2D. For patients with low-risk scores, the expression of PR18, PVRIG, and TNNI3K was upregulated, and DCAF13, FAM163A, LRP10, SGCB, and S100A9 were expressed at low levels.

### Comparing the survival prediction power of the PCG signature with other models

**Figure 3:**
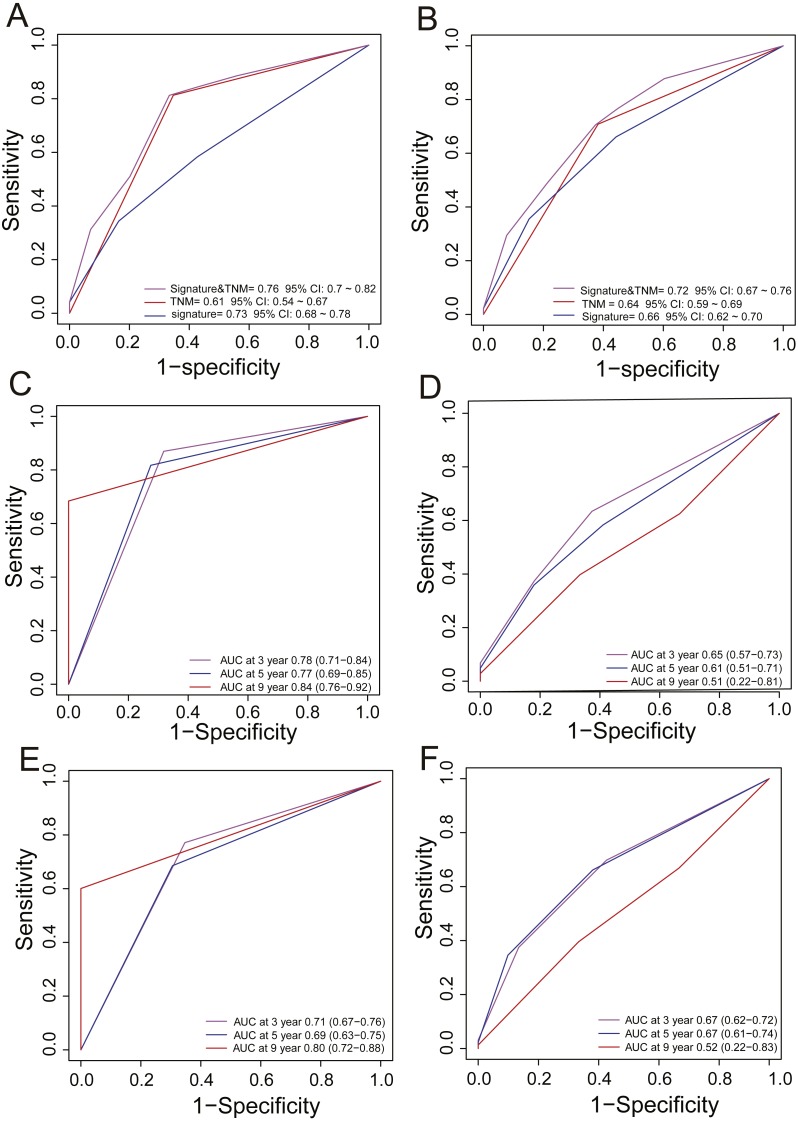
ROC analysis for comparing survival prediction power between the PCG signature and TNM stage in the training (A) and entire dataset (B) and time-dependent ROC analysis of the signature and TNM stage in the training (C, D) and entire dataset (E, F).

In clinical practice, TNM stage is an available prognostic biomarker routinely used as a non-invasive method for predicting the survival of HCC patients. To compare the survival prediction power of TNM stage and the signature, we performed ROC analysis. In the training dataset, the AUCs of the PCG signature were larger than the TNM stage (Signature-AUC Training = 0.73, 95% CI [0.68–0.78] *vs.* TNM-AUC Training = 0.61, 95% CI [0.54–0.67], Fig. 3A). This result was further verified in both TCGA and GEO datasets (Signature-AUC Test = 0.66 *vs.* TNM-AUC Test = 0.64, *n* = 553, Fig. 3B). We also compared the survival predictive power at 3, 5 and 9 years of the PCG signature with TNM staging by TimeROC analysis in both the TCGA dataset and in the entire datasets. In TCGA, the respective AUCs of the PCG signature and TNM staging were 0.78 (0.71–0.84) and 0.65 (0.57–0.73) at 3 years, 0.77 (0.69–0.85) and 0.61 (0.51–0.71) at 5 years and 0.84 (0.76–0.92) and 0.51 (0.22–0.81) at 9 years (Figs. 3C and 3D). In the entire TCGA and GEO dataset, the respective AUCs of the PCG signature and TNM staging were 0.71 (0.67–0.76) and 0.67 (0.62–0.72) at 3 years, 0.69 (0.63–0.75) and 0.67 (0.61–0.74) at 5 years and 0.80 (0.72–0.88) and 0.52 (0.22–0.83) at 9 years (Figs. 3E and 3F).

**Figure 4:**
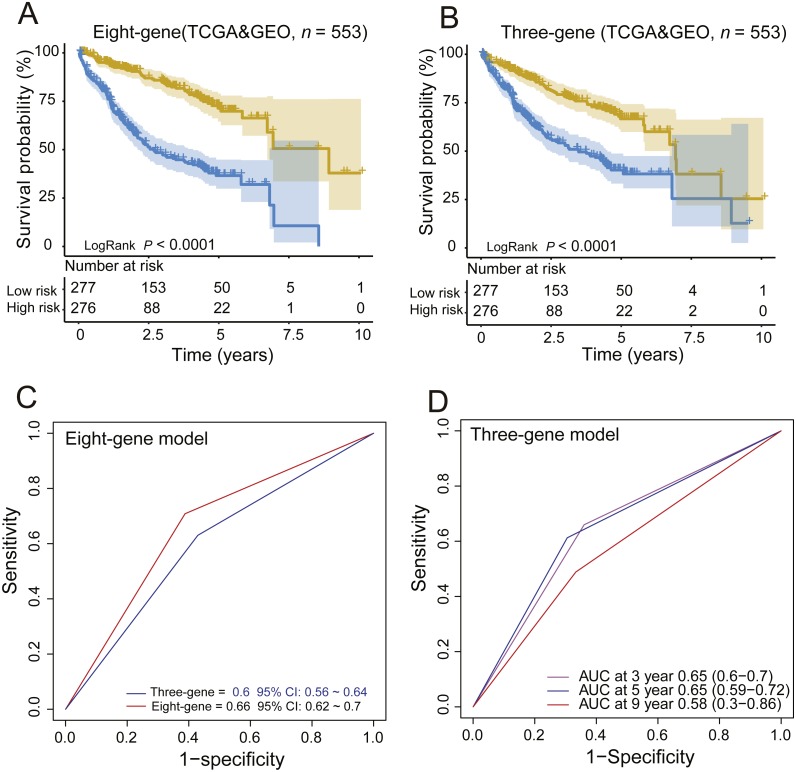
Comparing the survival prediction power of PCG signature with three-gene signature by Kaplan–Meier (A, B) and ROC (C) analysis and time-dependent ROC analysis of the three-gene signature in the entire dataset (*n* = 553).

Moreover, we also compared our eight-gene model with the three-gene prognostic signature (0.384 × RTN3 − 0.561 × SOCS2 − 0.434 × UPB1) (Li et al., 2017) in the entire TCGA and GEO dataset (*n* = 553), as they were all composed of PCGs. KM analysis showed the two models had good ability in stratification of HCCs (log-rank *P* < 0.0001); however, our eight-gene signature performed better, as it could divide the 553 HCCs into high-risk and low-risk groups (Fig. 4A), unlike the three-gene model, at more than 7.5 years (Fig. 4B). ROC analysis suggested that our signature outperformed the three-gene model in survival prediction (0.66, 95% CI: 0.62 ∼0.7 *vs.* 0.6, 95% CI: 0.56 ∼0.64, Fig. 4C). In addition, by TimeROC analysis, we compared the survival predictive power of the three- and eight-gene signatures at 3, 5 and 9 years, showing similar results to those above (Figs. 3E and 3F).

### The PCG signature is an independent prognostic factor

To assess whether the PCG signature was an independent risk factor for survival prediction, we performed a Chi-squared test and found no association between the PCG signature with a series of clinical parameters in the training or test groups (Table 3). Then, univariate Cox and multivariate Cox regression analyses were performed in the training dataset and showed that the PCG signature was an independent prognostic factor after adjusting for other clinical features, including sex, age and pTNM (high-risk group *vs.* low-risk group, HR = 7.23, 95% CI [4.19–12.47], *P* < 0.001, *n* = 332, Table 3). The same result was observed in the GEO dataset when adjusting for other clinical features, including sex, age ALT, AFP, cirrhosis, BCLC staging, CLIP staging and TNM staging (high-risk group *vs.* low-risk group, HR = 1.69, 95% CI [1.06–2.70], *P* = 0.03, *n* = 221, Table 3).

**Table 3:** Univariable and multivariable Cox regression analysis of the PCG signature and survival of HCC patients in the training and test group.

		**Univariable analysis**				**Multivariable analysis**			
**Variables**		**HR**	**95% CI of HR**		***P***	**HR**	**95% CI of HR**		***P***
			**lower**	**upper**			**lower**	**upper**	
TCGA dataset (*n* = 332)									
Age	>62 vs. ≤62	1.45	0.98	2.13	0.06	1.23	0.81	1.85	0.33
Sex	Male vs. Female	0.75	0.51	1.1	0.14	0.76	0.5	1.15	0.2
pTNM stage	I, II vs. III, IV	1.49	1.2	1.86	<0.001	1.11	0.71	1.73	0.66
PCG-signature	High risk vs. low risk	7.19	4.36	11.86	<0.001	7.23	4.19	12.47	<0.001
GSE14520 set (*n* = 251)									
Age	>62 vs. ≤62	0.99	0.97	1.01	0.40	0.99	0.97	1.02	0.56
Sex	Male vs.Female	1.70	0.82	3.52	0.15	1.14	0.54	2.40	0.73
ALT	>500 U.L vs. ≤500 U.L	1.08	0.70	1.66	0.73	0.71	0.45	1.12	0.14
AFP	>300 ng/ml vs. ≤300 ng/ml	1.63	1.06	2.50	0.03	0.69	0.35	1.37	0.29
Cirrhosis	Yes vs. No	4.62	1.14	18.80	0.03	4.80	1.15	20.09	0.03
BCLC stage	B,C vs.0,A	2.10	1.66	2.66	<0.001	1.63	1.05	2.53	0.03
CLIP stage	>2 vs. ≤1	1.91	1.55	2.36	<0.001	1.49	0.98	2.28	0.06
pTNM stage	III, IV vs. I, II	1.39	1.23	1.58	<0.001	1.01	0.83	1.24	0.91
PCG-signature	High risk vs. low risk	1.87	1.21	2.88	<0.001	1.69	1.06	2.70	0.03

### Stratification analysis

**Figure 5:**
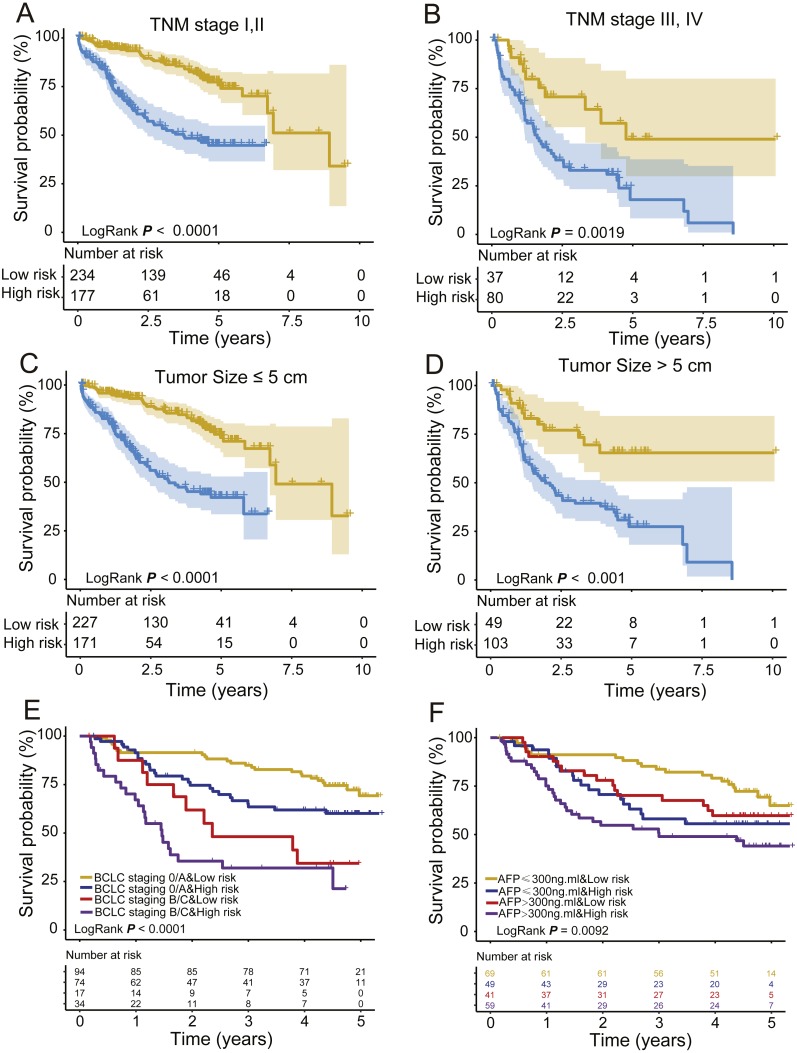
Stratified analysis of TNM (A, B) and T (C, D) low/high stage of the signature in the entire group and BCLC staging (E) and AFP (F) in the GEO dataset.

To obtain a better understanding of the clinical significance of the signature in HCC patients, we correlated the signature with a series of parameters in the two groups (*n* = 332). As seen in Tables 3 and 4, there was an association between the signature and TNM stage or pathologic T stage (tumour size) in the TCGA and GEO datasets (Chi-square test *P* < 0.05, Tables 4 and 5). Then, we integrated the TCGA and GEO datasets together and stratified the TNM stage and T stage by the PCG signature risk score. Kaplan–Meier curves showed that patients of low TNM stage (TNM I + II, *n* = 411) or high TNM stage (TNM III + IV, *n* = 117) and tumour size ≤ 5 cm (T stage I, II) or tumour size >5 cm (T stage III, IV) were further stratified into two different risk subgroups. The log-rank test showed that the high-risk patients of TNM or T low stage subdivided by the signature had shorter survival than the low-risk patients (log-rank test *P* < 0.001, Figs. 5A and 5C). Similarly, the TNM or T high stage patients were also divided into a high-risk group with lower OS and a low-risk group with higher OS (log-rank test *P* = 0.0019/< 0.001, Figs. 5B and 5D). In addition, the GEO set also showed an association between the signature and BCLC staging or AFP; KM analysis indicated that the HCCs could be subgrouped into four clusters by the signature and BCLC staging or alpha fetoprotein (AFP) with different OS: BCLC staging 0/A and low risk, BCLC staging B/C and low risk, BCLC staging 0/A and high risk, and BCLC staging B/C and high risk (log-rank test *P* < 0.001, Fig. 5E); or AFP ≤ 300 ng/ml and low risk, AFP > 300 ng/ml and low risk, AFP ≤ 300 ng/ml and high risk, and AFP > 300 ng/ml and high risk (log-rank test *P* = 0.0092, Fig. 5F).

**Table 4:** Association of the PCG signature with clinicoPathological characteristics in TCGA HCC patients.

**Variables**	**Training group**		***P***
	Low risk^*^	High risk^*^	
Age			0.38
>62	90	80	
≤62	77	85	
Sex			1.00
Female	55	55	
Male	112	110	
TNM staging			<0.001
I, II	137	104	
III, IV	23	45	
UNKNOWN	12	11	
Pathologic M			0.21
M0	123	117	
M1	0	3	
UNKNOWN	44	45	
Pathologic N			0.12
N0	173	54	
N1	3	0	
UNKNOWN	46	56	
Pathologic T			<0.001
T1-2	141	117	
T3-4	24	48	
UNKNOWN	2	0	

**Notes.**

* The Chi-squared test *P* value <0.05 was considered significant.

**Table 5:** Association of the PCG signature with clinicoPathological characteristics in GSE14520 HCC patients.

**Variables**	**Test group**		***P***
	Low risk^*^	High risk^*^	
Age			0.29
>62	92	96	
≤62	20	13	
Sex			0.09
Female	20	10	
Male	92	99	
TNM staging			<0.001
I, II	97	73	
III, IV	14	35	
UNKNOWN	2	0	
Tumor Size			<0.001
>5 cm	25	55	
≤5 cm	86	54	
UNKNOWN	1	0	
BCLC staging			0.02
0&A	94	74	
B&C	17	34	
UNKNOWN	1	1	
CLIP staging			0.08
>2	3	10	
≤2	108	98	
UNKNOWN	1	1	
HBV viral status			0.90
AVR-CC	27	29	
CC	81	75	
N	3	3	
UNKNOWN	1	2	
AFP (300 ng.ml)			0.03
>300 ng	41	59	
≤300 ng	69	49	
UNKNOWN	2	1	
ALT (50 U.L)			0.12
>50 U.L	40	51	
≤ 50 U.L	72	58	

**Notes.**

* The Chi-squared test *P* value <0.05 was considered significant.

### Functional characterization of the selected prognostic PCGs

**Figure 6:**
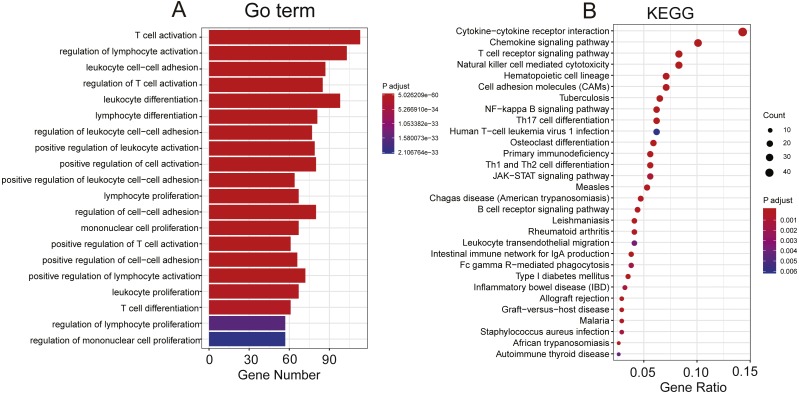
Functional enrichment of the co-expressed protein-coding genes with prognostic eight PCGs. Significantly enriched GO terms (A) and KEGG pathways (B) of the co-expressed protein-coding genes with the eight prognostic PCGs.

To explore the functional implications of these selected eight PCGs, we performed Pearson correlation analyses between the eight PCGs and protein-coding genes based on their expression levels in the TCGA and GEO datasets. A total of 776 protein-coding genes were highly correlated with at least one of the selected PCGs (Pearson correlation coefficient >0.5/≤0.5, *P*< 0.05). GO and KEGG of these co-expressed protein-coding genes were performed and revealed that co-expressed protein-coding genes in the two datasets were significantly enriched in 878 different terms, including 42 KEGG and 836 Go terms (*P* < 0.05). Most were immunity related and showed the eight genes might be involved in the immune system process through interacting with those co-expressed protein-coding genes that affect important biological processes such as T cell receptor signalling pathway, T cell activity and chemokine signalling pathway (Fig. 6).

## Discussion

Hepatocellular carcinoma (HCC) is a highly heterogeneous disease in terms of prognosis, as hepatocellular carcinoma patients with similar TNM stage have different survival times. As liver cancers are increasingly discovered and treated at an early stage, traditional clinicopathological indicators such as tumour size, vascular invasion, portal vein tumour thrombus and TNM stage have become difficult to adapt to the current needs of prediction of individual outcomes. In the era of precision medicine, screening prognostic molecular markers that fully reflect the biological characteristics of tumours is significant for individualized prevention and treatment of HCC patients. In the present study, we analysed the expression profiles of 553 pancreatic carcinoma samples from TCGA and GEO (Calderaro et al., 2017; Roessler et al., 2010; Roessler et al., 2012) and identified a robust eight-gene classifier associated with OS independent of clinical factors.

The successful development and wide clinical use of Oncotype DX (Bhutiani et al., 2018; Siow et al., 2018; Wang et al., 2018) (a distant disease recurrence score by analysing the expression of 21 genes) in breast cancer and Coloprint (Kopetz et al., 2015; Maak et al., 2013; Tan & Tan, 2011) (an 18-gene expression signature) in colon cancer showed that gene expression profiling has become the most promising high-throughput molecular approach for identifying new prognostic markers in cancers. Similar gene expression profiles have been developed for HCC in recent years. Ning Li et al. identified a HCC signature including 15 hub genes from the gene expression profile of 40 HCC samples (Li, Li & Chen, 2018). A study identified a three-gene prognostic signature from the GSE14520 dataset (Yang et al., 2018). Hoshida et al. discovered and validated a gene-expression signature associated with survival based on the profiles of liver tissue adjacent to the tumour in 306 HCC patients but failed to identify an outcome-associated signature from the tumor tissue profiles (Hoshida et al., 2008). However, these studies have limitations. Some studies lacked sufficient samples, and some failed to perform validation in independent datasets. Our study overcame these problems by collecting 553 HCC patients and validating the PCG signature obtained from the TCGA database in a GEO dataset produced from a different platform.

Through bioinformatics analyses, we found an eight-gene signature that divided HCC patients into low-risk and high-risk groups with significantly different survival times in the training or test dataset, demonstrating its good prognostic performance. Then, multivariate Cox regression analysis validated the power of the selected PCG signature in predicting OS in HCC patients independent of clinical features, such as age, sex and TMN stage. Moreover, the superior prediction ability of the eight-gene signature was confirmed by comparing with the three-gene model (Li et al., 2017) and the TNM stage, which is considered the traditional prognostic factor. The stratification analysis found that the PCG signature could subdivide HCC patients with TNM stage, T stage, BCLC stage and AFP, implying the robust prognostic power of the signature to serve as an auxiliary marker with those staging models.

In the PCG signature, high expression of DCAF13, FAM163A, LRP10, SGCB and S100A9 was associated with short survival time (univariate Cox coefficient >0), indicating these genes were risk factors for HCC patients. In contrast, GPR18, PVRIG and TNNI3K were protective factors. DCAF13 (DDB1 and CUL4 associated factor 13) is a protein coding gene located on 8q22.3 that encodes a protein containing five WD40 domain repeats in the central region and a C-terminal SOF1 domain (Chen et al., 2018). Although the function of DCAF13 has not yet been demonstrated, findings indicate that DCAF13 is associated with worse prognosis of breast cancer patients (Chin et al., 2007) and HCC patients (Cao et al., 2017), which is consistent with our finding. FAM163A (family with sequence similarity 163 member A), also known as NDSP (neuroblastoma-derived secretory protein), is found on chromosome 1q25.2 and encodes a 167 amino acid protein with a putative signal peptide (Vasudevan et al., 2009). NDSP is overexpressed in neuroblastoma tissue compared with normal tissues and may serve as a predictor of outcomes in neuroblastoma (Vasudevan et al., 2007). LRP10 (LDL receptor related protein 10) is involved in apolipoprotein internalization (Brodeur et al., 2012). However, a study mined the expression of thirteen LRPs from The Cancer Genome Atlas (TCGA) in ten common solid malignancies and found that LRP10 was significantly associated with decreased patient survival in three different malignancies: hepatocellular carcinoma, lung adenocarcinoma, and pancreatic adenocarcinoma (Gonias et al., 2017). Our finding confirms the potential role LRP10 in HCC prognosis and another reported three-gene prognostic signature for HCCs (Li et al., 2017). S100A9 (S100 calcium binding protein A9) belongs to a family of 25 homologous low-molecular-weight intracellular calcium-binding proteins. S100A9 expression is significantly higher in a variety of tumours compared to normal tissues or healthy individuals, and may be a potential marker for poor prognosis of cancer patients (Gunaldi et al., 2015; Huang et al., 2018; Shabani et al., 2018; Yun et al., 2015). SGCB (sarcoglycan beta), a member of the sarcoglycan family, is associated with limb-girdle muscular dystrophy. There is no research on the role of SGCB in cancer. GPR18 (G protein-coupled receptor 18) is a member of the G-protein-coupled receptor (GPCR) family. GPCRs are involved in regulating important biological functions, including cellular motility, growth and differentiation, and gene transcription, and play a key role in cancer progression. Qin et al., (2011) demonstrated that GPR18 was overexpressed in melanoma metastases and may be highly relevant for the malignant behaviour of melanoma. However, in our study, GPR18 was identified as a protective factor for HCC patients. PVRIG (PVR related immunoglobulin domain containing), also known as CD112R, is a member of the poliovirus receptor–like proteins, preferentially expressed on T cells and NK cells (Zhu et al., 2016). Blockade of CD112R enhanced trastuzumab-triggered antitumour response by human NK cells and could be used to treat breast cancer (Xu et al., 2017). The third protective factor TNNI3K (troponin I-interacting kinase) is a cardiac-specific kinase whose biological function remains largely unknown. Although we inferred the function of these PCGs by GO and KEGG analyses and validated their expression in six cell lines, the biological roles of the selected 8 genes in immunity are still not clear and should be investigated in further experimental studies.

## Conclusions

We have identified an eight-gene signature associated with the survival of hepatocellular carcinoma patients in a large HCC cohort. As a result, this signature could be used as a promising prognostic tool to achieve risk stratification of patients with HCC in the clinic and could serve as an auxiliary marker with existing staging models, such as TNM stage and T stage, which have been validated in more than 500 HCCs.

##  Supplemental Information

10.7717/peerj.6548/supp-1 Table S1 PCGs of Univariate Cox regression analysis in the TCGA set (*P* < 0.05, *n* = 332)

10.7717/peerj.6548/supp-2 Table S2 The signature composed of PCGs in the training datasets (*n* = 332)

10.7717/peerj.6548/supp-3 Table S3 Co-expression gene with the 8 genes in TCGA and GEO dataset

10.7717/peerj.6548/supp-4 Table S4 GO and KEGG analysis

## Abbreviations

PCG: protein coding gene
ROC: receiver operating characteristic
AUC: area under the ROC curve
OS: overall survival
HCC: hepatocellular carcinoma
SD: standard deviation
TCGA: The Cancer Genome Atlas

## Additional Information and Declarations

### Data Availability

The following information was supplied regarding data availability: The dataset with mRNA expression profiling and clinical information was taken from GEO: GSE14520. The dataset including gene expression profile and associated corresponding clinical information of patients analysed in this study was downloaded from The Cancer Genome Atlas (TCGA, http://cancergenome.nih.gov/): Liver cancer data: https://xenabrowser.net/datapages/?cohort=GDC%20TCGA%20Liver%20Cancer%20(LIHC)
FPKM: https://gdc.xenahubs.net/download/TCGA-LIHC/Xena_Matrices/TCGA-LIHC.htseq_fpkm.tsv.gz
Clinical data, survival data: https://gdc.xenahubs.net/download/TCGA-LIHC/Xena_Matrices/TCGA-LIHC.survival.tsv.gz
Phenotype: https://gdc.xenahubs.net/download/TCGA-LIHC/Xena_Matrices/TCGA-LIHC.GDC_phenotype.tsv.gz.
